# *Usp26* mutation in mice leads to defective spermatogenesis depending on genetic background

**DOI:** 10.1038/s41598-019-50318-6

**Published:** 2019-09-24

**Authors:** Kohei Sakai, Chizuru Ito, Mizuki Wakabayashi, Satoko Kanzaki, Toshiaki Ito, Shuji Takada, Kiyotaka Toshimori, Yoichi Sekita, Tohru Kimura

**Affiliations:** 1 Laboratory of Stem Cell Biology, Department of Biosciences, Kitasato University School of Science, 1-15-1 Kitasato, Minami-ku, Sagamihara, Kanagawa 252-0373 Japan; 2 Department of Functional Anatomy and Reproductive Biology and Medicine, Graduate School of Medicine, 1-8-1, Inohana, Chuo-ku, Chiba, Chiba, Japan; 3 Department of Systems BioMedicine, National Research Institute for Child Health and Development, 2-10-1, Okura, Setagaya-ku, Tokyo 157-8535 Japan; 4 Future Medicine Research Center, Chiba University Chiba University, 1-8-1, Inohana, Chuo-ku, Chiba, Chiba 260-8670 Japan

**Keywords:** Development, Spermatogenesis

## Abstract

Spermatogenesis is a reproductive system process that produces sperm. Ubiquitin specific peptidase 26 (USP26) is an X chromosome-linked deubiquitinase that is specifically expressed in the testes. It has long been controversial whether *USP26* variants are associated with human male infertility. Thus, in the present study, we introduced a mutation into the *Usp26* gene in mice and found that *Usp26* mutant males backcrossed to a DBA/2 background, but not a C57BL/6 background, were sterile or subfertile and had atrophic testes. These findings indicate that the effects of the *Usp26* mutation on male reproductive capacity were influenced by genetic background. Sperm in the cauda epididymis of *Usp26* mutant mice backcrossed to a DBA/2 background were decreased in number and showed a malformed head morphology compared to those of wild-type mice. Additionally, histological examinations of the testes revealed that the number of round and elongated spermatids were dramatically reduced in *Usp26* mutant mice. The mutant mice exhibited unsynapsed chromosomes in pachynema and defective chiasma formation in diplonema, which presumably resulted in apoptosis of metaphase spermatocytes and subsequent decrease of spermatids. Taken together, these results indicate that the deficiencies in fertility and spermatogenesis caused by mutation of *Usp26* were dependent on genetic background.

## Introduction

Spermatogenesis is a complex system of differentiation that involves the self-renewal of spermatogonial stem cells, the differentiation of spermatogonia into meiotic spermatocytes, and morphogenesis of haploid spermatids^1^. These differentiation processes are strictly regulated by transcriptional, translational, and post-translational mechanisms, and alterations in these processes may be causative factors for male infertility and subfertility^2^. Thus, the processes of spermatogenesis have been the subjects of extensive study in the fields of reproductive biology and medicine.

Ubiquitination is a fundamental biological process that controls the stability and degradation of cellular proteins^3^. The addition of ubiquitin to substrate proteins is mediated by the relayed reactions of ubiquitin-activating enzymes (E1s), ubiquitin-conjugating enzymes (E2s), and ubiquitin ligases (E3s), and promotes the degradation of target proteins in proteasomes. In contrast, the removal of ubiquitin from substrate proteins is catalyzed by ubiquitin-specific peptidases (USPs; also known as deubiquitinases [DUBs]) and prevents target proteins from being degraded^4^. The balance between ubiquitination and deubiquitination regulates a wide variety of biological processes, including spermatogenesis^5,6^. Although the mammalian genome encodes a large number of E3 ubiquitin ligases and USPs, their roles in physiology and pathology remain to be elucidated.

*Usp26*/*USP26*, which is located on the X chromosome, is thought to be a retrogene that originates from autosomal *Usp39*/*USP39* and is specifically expressed in the testes in both mice and humans^7–10^. Several substrates of USP26, such as the androgen receptor, MDM2, SMAD7, and polycomb repressive complex 1 (PRC1), have been identified^11–15^, and a substantial number of studies have reported associations between *USP26* single nucleotide polymorphisms (SNPs) and human male infertility^16–23^. However, other studies have provided evidence that does not support a direct association between *USP26* variants and male infertility^24–30^. Furthermore, of the 18 known human variants of this gene, 17 show the same level of deubiquitinase activity as wild-type USP26 *in vitro*^31^. Additionally, the recent two studies of *Usp26* mutant mice also reported the conflicting results^32,33^; one group reported that their *Usp26* mutant mice displayed subfertility associated with abnormal spermatogenesis whereas another group found no abnormality in fertility and spermatogenesis in their *Usp26* KO mice. Thus, the role of *USP26* variants and *Usp26* mutations as risk factors for male infertility remains controversial, and it is possible that other genetic factors may contribute in a cooperative manner to the phenotype of *USP26* variants and *Usp26* mutations in humans and mice.

In the present study, mice with a mutation of *Usp26* were generated and then backcrossed to either a DBA/2 background or a C57BL/6 background to investigate genetic effects. The *Usp26* mutant males that were backcrossed on a DBA/2 background were sterile or subfertile and had smaller testes than wild-type mice, whereas *Usp26* mutant males backcrossed on a C57BL/6 background exhibited normal fertility and testis size. The mutant males backcrossed on a DBA/2 background also displayed defective spermatogenesis that resulted in fewer and malformed sperm. The present results clearly demonstrate that the effects of the *Usp26* mutation on murine spermatogenesis were dependent on genetic background, which may account for the conflicting findings regarding the relationship between *USP26* variants/*Usp26* mutations and male infertility.

## Results

### Generation of Usp26 mutant mice

**Figure 1:**
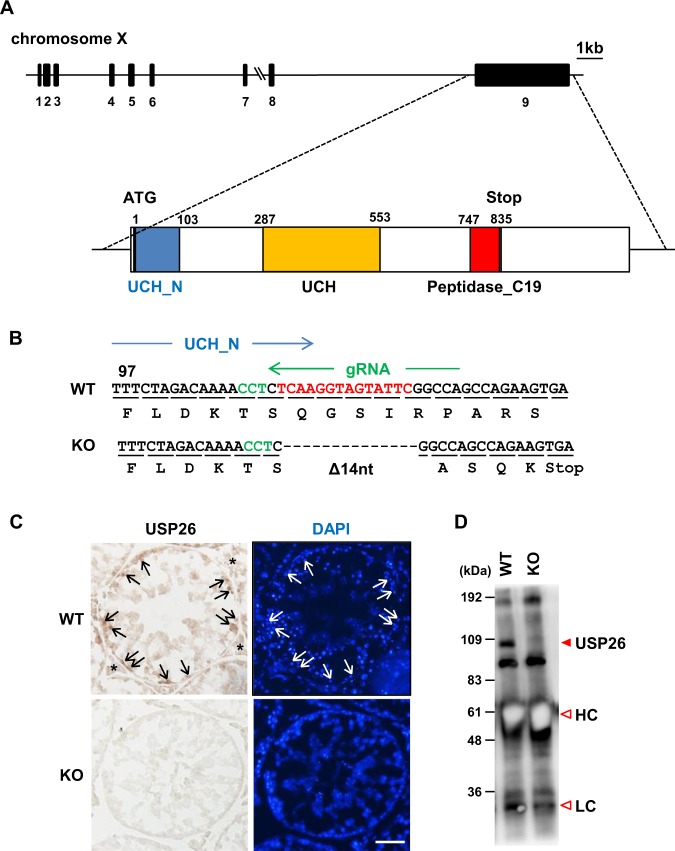
Generation of *Usp26* mutant mice. (**A**) Structure of the mouse *Usp26* gene. The USP26 protein is encoded by a single exon, exon 9. The domain structures of the Usp26 protein are shown at the bottom. (**B**) The mutation introduced into the *Usp26* gene. Using the CRISPR/Cas9 system, a 14-nucleotide deletion was introduced into zygotes that caused a frame shift and premature translational termination in the N–terminal ubiquitin hydroxylase domain (UHC_N). The deleted nucleotides and PAM sequence are shown in red and green letters, respectively. (**C**) Immunohistochemistry of the testes using an anti-USP26 antibody Ab1. Nuclei are counterstained with DAPI. Strong signals were detected in spermatogonia, whereas weaker signals were detected in spermatids and Leydig cells. Arrows and asterisks indicate spermatogonia and Leydig cells, respectively. The mice backcrossed to a DBA/2 background 5 generations were analysed. Bars: 50 µm. (**D**) Western blot analysis following immunoprecipitation (IP-WB). The USP26 protein in testes lysates was immunoprecipitated by anti-USP26 antibody Ab2 and then subjected to Western blot analysis suing the same antibody. USP26 protein was not detectable in the testes of *Usp26* mutant mice. HC, heavy chain; LC, light chain.

To investigate the functions of *Usp26* related to male mouse reproduction, a mutation was introduced into the *Usp26* locus using the CRISPR/Cas9 system. Although the *Usp26* gene is composed of nine exons, its open reading frame (ORF) is encoded by a single exon (exon 9; Fig. 1A), since *Usp26* gene was generated by the retrotransposition of the autosomal *Usp37* gene. Fertilized eggs collected from mating pairs of B6D2F1 mice, F1 hybrids crossed between C57BL/6 females and DBA/2 males, were electroporated with *Cas9* mRNA and gRNA corresponding to the N-terminal of ubiquitin carboxyl-terminal hydroxylase domain (UCH_N; Fig. 1A,B) and then transferred to pseudopregnant females. A heterozygous F0 female (*Usp26*^+/*Mut*^) underwent the deletion of 14 nucleotides within the *Usp26* ORF, which resulted in premature translational termination (Fig. 1B). This female was then crossed with C57BL/6 males to produce *Usp26* mutant males (*Usp26*^*Y*/*Mut*^) on a mixed genetic background between the C57BL/6 and DBA/2 strains.

*Usp26* mRNA is abundantly expressed in spermatogonia, but this expression declines after meiotic entry and becomes undetectable in pachytene spermatocytes^8,9^; weak expression resumes in round spermatids^9^. Consistent with these findings, immunohistochemical analyses revealed that USP26 was strongly expressed in the spermatogonia of wild-type mice (Fig. 1C). The weak but consistent USP26 signals were detectable in spermatids and Leydig cells, as shown in previous immunohistochemical analyses^7,10^. No USP26 signals were detected in the testes of *Usp26* mutant mice (Fig. 1C). Western blot analysis following immunoprecipitation of USP26 protein also showed that USP26 was undetectable in the testis of *Usp26* mutant mice (Fig. 1D).

### Sterility or subfertility in Usp26 mutant mice

**Figure 2:**
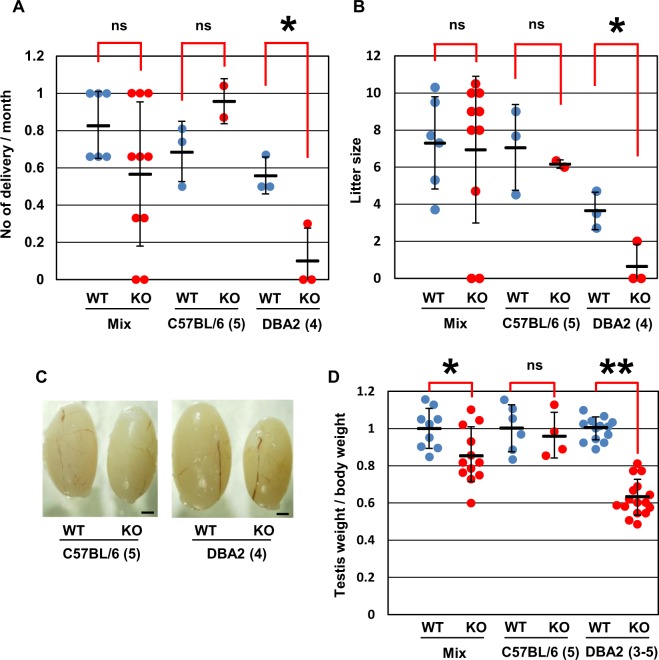
Reproductive ability of *Usp26* mutant mice. (**A**,**B**) Number of deliveries per month (**A**) and number of pups per delivery (**B**). Wild-type and *Usp26* mutant males on different genetic backgrounds were crossed with wild-type females. The number of deliveries and litter size were both significantly reduced in *Usp26* mutant mice backcrossed on a DBA/2 background (Student’s *t*-test; *P < 0.05; ns). The mean ± standard deviation is shown. (**C**) Testes of *Usp26* mutant mice that were backcrossed with C57BL/6 (left) or DBA/2 mice (right). Bars: 1 mm. (**D**) Ratio of testis weight versus body weight. The average of wild-type mice is given as 1. The values of individual mice and the mean ± standard deviation are shown; the ratio was significantly reduced in mutants backcrossed on a DBA/2 background (Student’s *t*-test; *P < 0.05, **P < 10^−9^; ns). The number of backcross generations are shown in the parentheses.

**Figure 3:**
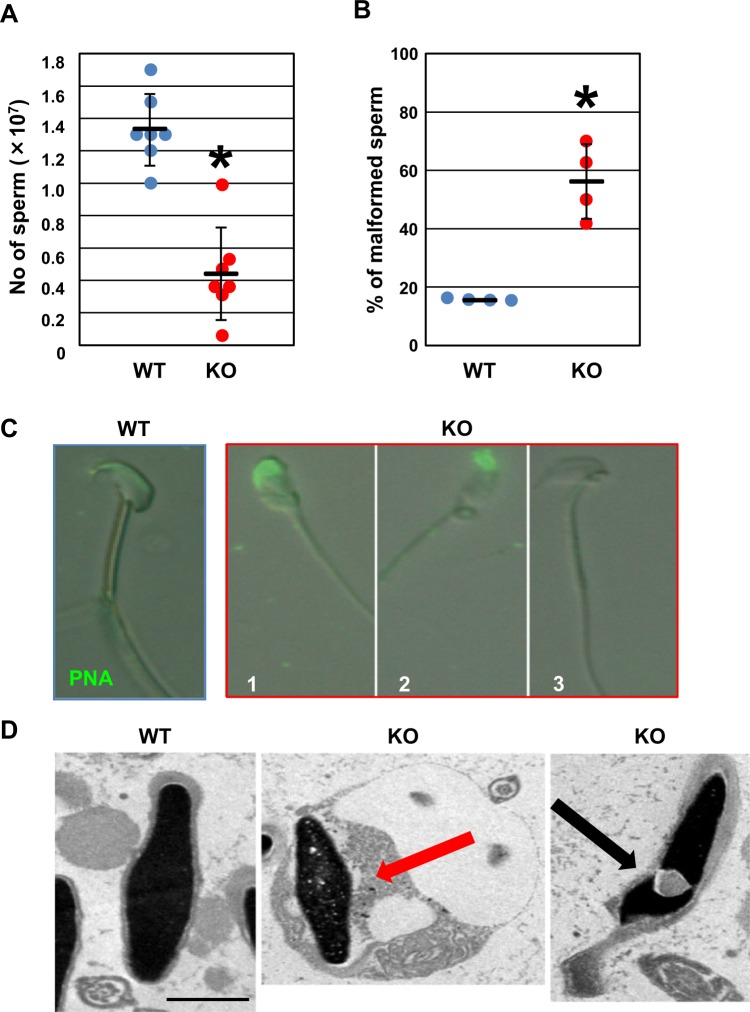
Sperm from *Usp26* mutant mice. (**A**) Number of sperm in the cauda epididymis. The number of sperm in the cauda epididymis was significantly reduced in *Usp26* mutant mice that were backcrossed to a DBA/2 background 3–4 generations (Student’s *t*-test; *P < 10^−4^). The mean ± standard deviation is shown. (**B**) Percentages of morphologically abnormal sperm in the cauda epididymis. The percentages of malformed sperm were significantly elevated in mutant mice (Student’s *t*-test; ^*^P < 0.01). The mean ± standard deviation is shown. The mice backcrossed to a DBA/2 background 4 generations were used in (**B**–**D**). (**C**) Representative photos of malformed sperm. The sperm were stained with PNA (green). The sperm of *Usp26* mutant mice showed an abnormal head morphology (1, 2) and a lack of acrosomes (3). (**D**) Electron microscopic analysis. The nuclei of sperm in the mutant mice were surrounded by residual cytoplasm (middle, red arrow) and invaginated with cytoplasm (right, black arrow). Bar: 1 µm.

**Figure 4:**
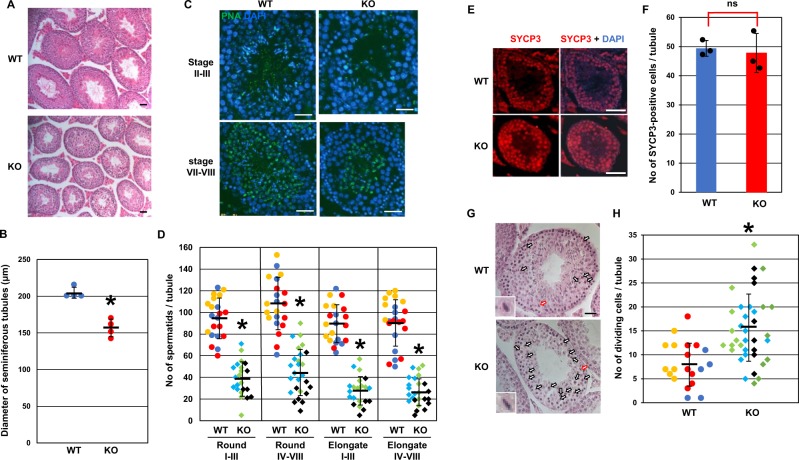
Spermatogenesis in *Usp26* mutant mice. (**A**) HE–stained sections of adult testes. Bars: 100 µm. The mice backcrossed to a DBA/2 background 4 generations were used in Fig. 4. (**B**) Diameter of the seminiferous tubules. A total of 20 tubules were randomly selected from each mouse and the diameters were measured. The diameters were significantly reduced in *Usp26* mutant mice (Student’s *t*-test; *P < 0.001); the mean ± standard deviation is shown. (**C**) Sections of stage II–III and stage VII–VIII tubules stained with PNA and DAPI. Bars: 50 µm. (**D**)Numbers of round and elongating/elongated spermatids in stage I–III and stage IV–VIII tubules. A total of 8–10 tubules were analysed per mouse (wild-type, *n* = 3; mutant, *n* = 3). The numbers of spermatids in the individual tubules were plotted and the values for each mouse are represented in different colors. The numbers of round and elongating/elongated spermatids were significantly reduced in mutant mice (Student’s *t*-test; *P < 10^−12^). The mean ± standard deviation is shown. (**E**) Sections of testes stained with anti–SYCP3 antibody (red); the nuclei were stained with DAPI. Bars: 50 µm. (**F**) Number of spermatocytes in meiotic prophase per tubule. Five tubules were randomly selected from each mouse and the number of S SYCP3–positive cells was counted (Student’s *t*-test; ns); the mean ± standard deviation is shown. (**G**) HE–stained sections of stage XII tubules. Arrows represent germ cells in meiotic cell division and the inserts show higher magnification views of dividing cells (red arrows). Bars: 20 µm. (**H**) Numbers of germ cells undergoing meiotic cell division in stage XII tubules. A total of 5–11 stage XII tubules were randomly selected from each mouse and analysed (wild-type, *n* = 3; mutant, *n* = 4). The numbers of dividing meiotic cells in the individual tubules were plotted and the values for each mouse are represented in different colors. The number of germ cells in meiotic cell division was significantly reduced in mutant mice (Student’s *t*-test; *P < 10^−4^); the mean ± standard deviation is shown.

**Figure 5:**
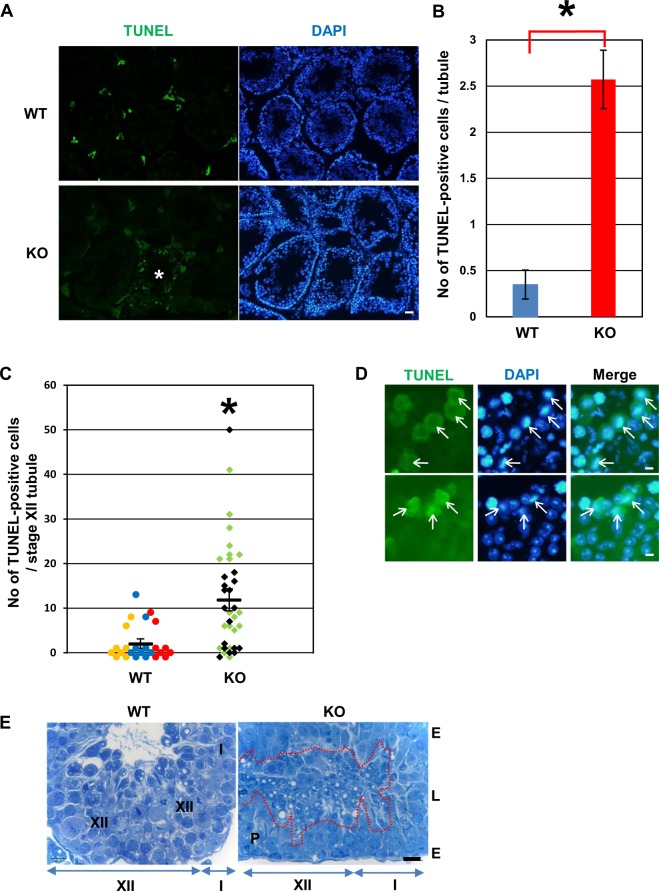
Apoptosis of germ cells in *Usp26* mutant mice. (**A**) TUNEL analysis of *Usp26* mutant testes. Apoptotic cells were detected with TUNEL assays (green) and the nuclei were stained with DAPI (blue). Asterisk represents seminiferous tubules containing TUNEL–positive cells. The mice backcrossed to a DBA/2 background 5 generations were used in Fig. 5A–D. Bars: 100 µm. (**B**) Average number of TUNEL–positive cells per tubule. More than 140 tubules were analyzed per mouse. Mutant mice exhibited a greater number of apoptotic cells (Student’s *t*-test; *P < 0.05). The mean ± standard deviation is shown. (**C**) The number of TUNEL–positive cells per stage XII tubule. The number of TUNEL–positive cells in the individual tubules were plotted and the values for each mouse are represented in different colors. The number of TUNEL–positive cells was significantly elevated in mutant mice (Student’s *t*-test; *P < 10^−4^); the mean ± standard deviation is shown. (**D**)Apoptotic cells in meiotic division in stage XII–I tubules in *Usp26* mutant mice. Arrows indicate TUNEL–positive metaphase spermatocytes. Bar: 5 µm. (**E**) Toluidine blue-stained stage XII–I seminiferous tubules of wild-type (transverse section) and *Usp26* mutant mice (longitudinal section). There was an accumulation of degenerated spermatids detached from the epithelial layer within the lumen of stage XII–I tubules in *Usp26* mutant mice (enclosed with red dotted line). The mice backcrossed to a DBA/2 background 4 generations were used. P, meiotic figure of the primary spermatocyte; E, seminiferous epithelium; L, lumen. Bar: 10 µm.

**Figure 6:**
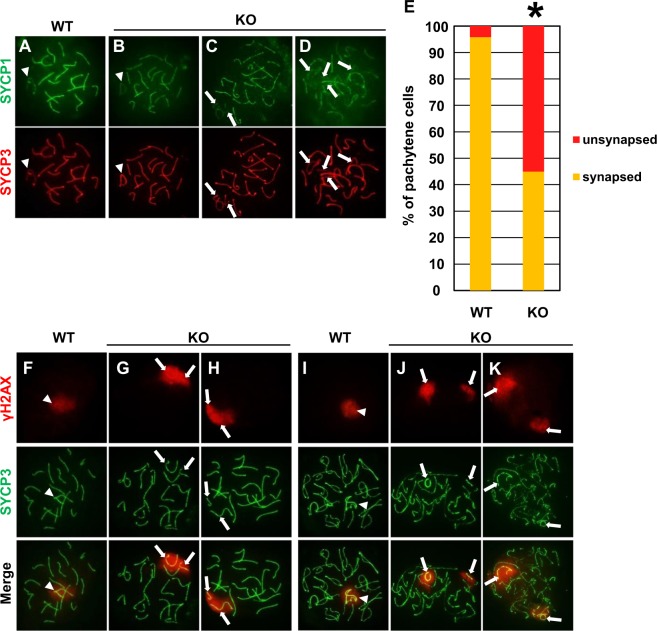
Synapsis and chiasma formation in pachynema and diplonema of *Usp26* mutant mice. (**A**–**D**) Synapsis in pachynema of wild-type (**A**) and *Usp26* mutant mice (**B**–**D**). The spreads were stained with antibodies against SYCP1 (green) and SYCP3 (red). Pachynema of the mutant mice included normally synapsed cells (**B**), the cells with unsynapsed sex chromosomes (**C**) and the cells with unsynapsed sex chromosomes and autosomes (**D**). Arrowheads (**A**,**B**) and arrows (**C**,**D**) indicate paired sex bodies and unsynapsed chromosomes, respectively. The mice backcrossed to a DBA/2 background 6 generations were used in Fig. 6. (**E**) The percentages of pachynema with unsynapsed chromosomes. The percentages were significantly increased in *Usp26* mutant mice (wild-type, 24 nuclei; mutant, 40 nuclei; *P* < 10^−4^ by *χ*^2^ test). (**F**–**H**) The γH2AX signals of pachynema of wild-type (**F**) and *Usp26* mutant mice (**G**,**H**). The spreads were stained with antibodies against γH2AX (red) and SYCP3 (green). The γH2AX signals were detected in sex bodies of wild-type mice (**F**) and unsynapsed sex chromosomes of mutants (**G**,**H**). Arrowheads (**F**) and arrows (**G**,**H**) indicate sex bodies and unsynapsed chromosomes, respectively. (**I**–**K**) The γH2AX signals in diplonema of wild-type (**I**) and *Usp26* mutant mice (**J**,**K**). The separate γH2AX-positive chromatin domains indicate failure of chiasma formation between the sex chromosomes (arrows in **J**,**K**). Arrowheads indicate sex bodies (**L**).

To examine reproductive ability, *Usp26* mutant males on a mixed background were crossed with control (wild-type and heterozygous) siblings or C57BL/6 female mice. Over 3 months, two of 10 mutant males did not sire any offspring at all during crosses with control siblings or C57BL/6 females, but six of the mutant mice sired 0.67–1 times per month, as did wild-type siblings (Fig. 2A,B). Because phenotypic variations may be attributable to a mixed genetic background, the heterozygous female mice were backcrossed with DBA/2 male mice 3–5 generations or C57BL/6 male mice 5 generations. The number of backcross generations are shown in the parentheses of the figures (Fig. 2) or described in the figure legends (Figs 1 and 3–6). The *Usp26* mutant males that were backcrossed to a DBA/2 background were infertile or subfertile during matings with DBA2 females over 6 months (Fig. 2A,B). In contrast, the mutant males backcrossed to a C57BL/6 background showed normal fertility during matings with C57BL/6 females. Consistent with this observation, the *Usp26* mutant mice backcrossed to a DBA/2 background had significantly smaller testes than wild-type mice, whereas those backcrossed to a C57BL/6 background did not (Fig. 2C,D).

### Spermatogenesis in Usp26 mutant mice

The above results prompted further examinations of sperm in the cauda epididymis and of spermatogenesis in the testes of *Usp26* mutant mice that were backcrossed on a DBA/2 background (Figs 3–6). The number of sperm in the cauda epididymis of *Usp26* mutant mice was significantly lower than that in wild-type mice (Fig. 3A). Sperm in *Usp26* mutant mice that were stained with peanut agglutinin (PNA), which binds to acrosomal lectin, exhibited malformed acrosomes or a lack of acrosomes (Fig. 3B,C). Additionally, electron microscopic analyses of sperm from *Usp26* mutant mice revealed that most of the sperm nuclei were encapsulated with residual cytoplasm (Fig. 3D, middle) and that some of the nuclei were invaginated by cytoplasm (Fig. 3D, right).

Next, spermatogenesis in adult testes was examined. The diameter of the seminiferous tubules in *Usp26* mutant mice was significantly smaller than that in wild-type mice (Fig. 4A,B). After the stage of the seminiferous tubules was determined by acrosomal and nuclear morphology, the number of spermatids during spermiogenesis was compared between wild type and *Usp26* mutant mice (Fig. 4C,D). In *Usp26* mutant mice, the number of both round and elongating/elongated spermatids was approximately half that of wild-type mice throughout spermiogenesis. In contrast, the number of SYCP3–positive spermatocytes in *Usp26* mutant mice was comparable to that of wild-type mice (Fig. 4E,F).

To confirm the decrease of spermatids in the mutant mice, expression of spermatocyte and spermatid markers was examined. In *Usp26* mutant mice, SYCP3 protein levels increased 2–3–fold (Fig. S1) and the *Sycp3* mRNA levels were slightly upregulated (Fig. S2). This increase may reflect the relative increase of spermatocytes, which was caused by the decrease of spermatids. Consistently, expression of the spermatid marker genes such as protamine (*Prm1*) and transition protein (*TP1* and *TP2*) was significantly decreased in *Usp26* mutant mice (Fig. S2). Unexpectedly, the protein levels of Equatorin, an acrosomal protein, were not altered between wild-type and mutant mice. Considering the morphological abnormalities of sperm (Fig. 3B–D), this is presumably due to the altered stability of the acrosomal proteins (Fig. S1). Taken together, these findings suggest that there was a decrease in the number of germ cells at the transition from spermatocytes to round spermatids in *Usp26* mutant mice. Additionally, the number of germ cells undergoing meiotic cell division in stage XII tubules significantly increased in *Usp26* mutant mice, which suggests a delay and/or arrest of meiotic cell division (Fig. 4G,H).

Next, TUNEL analyses were performed to determine whether the defective spermatogenesis was due to apoptosis. There was an increase in the average number of TUNEL–positive cells per seminiferous tubule in *Usp26* mutant mice (Fig. 5A,B), as well as variation in the numbers of TUNEL–positive cells among the tubules of mutant mice, which suggests that apoptosis occurred at specific stages of germ cell differentiation. Specifically, the number of TUNEL–positive cells was significantly elevated in stage XII tubules (Fig. 5C). The majority of TUNEL–positive cells were metaphase spermatocytes in stage XII–I tubules (Fig. 5D). Moreover, histological examination showed that degenerated spermatids consistently accumulated within the lumen of stages XII–I seminiferous tubules in *Usp26* mutant mice (Fig. 5E).

Increased apoptosis in metaphase spermatocytes is usually associated with a defect in crossover formation^34–37^. We next investigated whether *Usp26* mutant pachytene spermatocytes exhibited defects in synapsis formation by staining central and lateral elements of synaptonemal complexes with anti–SYCP1 and anti–SYCP3 antibodies, respectively (Fig. 6). In wild-type pachynema, 19 pairs of autosomes were fully synapsed, and X–Y chromosomes were partially synapsed in pseudoautosomal region (Fig. 6A). In contrast, in approximately a half of *Usp26* mutant pachynema, the majority of chromosomes were fully synapsed, but some chromosomes were not synapsed at all (Fig. 6B–D). In most cases, only X–Y chromosomes were unsynapsed (Fig. 6C) whereas in a small number of cells both sex chromosomes and autosomes were unsynapsed (Fig. 6D). The unsynapsed chromosomes resided in γH2AX-potisitive chromatin domains (Fig. 6F–H). The percentages of pachynema with unsynapsed chromosomes were significantly elevated in *Usp26* mutant mice (Fig. 6E). Remarkably, the homologous chromosomes of each bivalent, including sex chromosomes, were bound at chiasmata in wild-type diplonema (Fig. 6I) whereas the γH2AX-positive sex chromatin domains were completely separated in the mutant aberrant diplonema (Fig. 6J,K), which indicates that chiasmata were not formed between these chromosomes. In conclusion, these results show that the *Usp26* mutation resulted in defective spermatogenesis that was dependent on genetic background.

## Discussion

The present study demonstrated that mice carrying a mutation of the *Usp26* gene, when backcrossed on a DBA/2 background, but not a C57BL background, were sterile or subfertile (Fig. 2). This finding indicates that there is a strain-specific genetic modifier (or modifiers) that acts cooperatively with the *Usp26* mutation. Additionally, the present study determined that sterility or subfertility in the mutant mice was caused by defective spermatogenesis. Although several case studies have shown that *USP26* variants are associated with human infertility^16–23^, other reports have proposed that *USP26* variants are not directly associated with human infertility^24–30^. Considering the present results in mice, this controversy regarding the role of *USP26* in human fertility may be explained by the effects of genetic modifier(s) that might also be present in humans.

In the present study, sperm in the cauda epididymis of *Usp26* mutant mice backcrossed on a DBA/2 background were reduced in number and exhibited malformations that included abnormally shaped acrosomes, a lack of acrosomes, residual bodies around the nuclei, and protrusion of the cytoplasm into nuclei (Fig. 3). These results suggest that spermiogenesis was defective in *Usp26* mutant mice. Additionally, the number of germ cells consistently and sharply declined at the transition from meiotic spermatocytes to the early stages of round spermatids (Fig. 4). This change was accompanied by apoptosis of dividing germ cells as well as accumulation of degenerated round spermatids in the seminiferous lumen (Fig. 5). We also observed unsynapsed chromosomes in pachynema and defective chiasma formation in diplonema in the mutant mice (Fig. 6). It is likely that crossovers could not take place in the unsynapsed chromosomes of these mutant cells. Because meiotic metaphase arrest is typical of mutant mice that fail to generate crossovers^34–37^, these abnormalities likely resulted in apoptosis in metaphase spermatocytes. Although *Usp26* is abundantly expressed in spermatogonia, the USP26 protein has also been detected in early stage spermatocytes and spermatids^7–10^. More specifically, because USP26 is reportedly localized at the interface between germ cells and Sertoli cells^7^, aberrant interactions between germ cells and Sertoli cells may lead to defective spermatogenesis during and after meiotic cell division in *Usp26* mutant mice. Alternatively, USP26 may be involved in the formation of acrosomes because it has been shown that USP26 is detectable in the acrosomes of elongated spermatids^7^.

Several substrates of USP26, including androgen receptor (AR), MDM2, SMAD7, and PRC1, have been identified^11–15^. Because USP26 prevents the degradation of these substrates, their levels would be downregulated in *Usp26* mutant mice. Studies on the cell type-specific deletion of AR have shown that AR in germ cells is not required for proper spermatogenesis, whereas the deletion of AR in Sertoli cells, Leydig cells, and peritubular myoid cells results in defective spermatogenesis^38,39^. Intriguingly, Leydig cell-specific AR-deficient mice exhibit a decrease in haploid cells^40^, just as was observed in the *Usp26* mutant mice in the present study. Because USP26 expression is detectable in Leydig cells (Fig. 1C)^10^, these cells may be critical in the regulation of spermatogenesis by USP26. However, conditional deletion of *Usp26* in Leydig cells must be performed to clarify this issue. On the other hand, MDM2 is a ubiquitin ligase for TRP53, which is a tumor suppressor, and *Trp53*-deficient mice exhibit strain-specific spermatogenic defects^41^. Although mice carrying activated *Trp53* do not exhibit defective spermatogenesis, these previous analyses were carried out using mice with a mixed C57BL/6 and 129/Sv background^42^. Thus, studies in mice on a DBA/2 background are necessary.

A recent study found that the *Usp26* mutant mice backcrossed on a C57BL/6 background more than 6 generations exhibited subtle declines in litter size and an increased number of seminiferous tubules with defective spermatogenesis^32^. In contrast, in the present study, no obvious differences in fertility or spermatogenesis were observed in the *Usp26* mutant mice backcrossed on a C57BL/6 background 5 generations. Similarly, another recent study reported that no abnormality in spermatogenesis and fertility were observed in the *Usp26* mutant mice generated in F1 hybrids between C57BL/6 and CBA strains^33^. It is possible that the phenotypic differences between these *Usp26* mutants were due to differences in the number of backcross generations to a C57BL/6 background and/or in how the mutations were introduced; i.e., the replacement of exon 9 by a neomycin-resistance cassette versus frameshift mutations. Future studies to determine the cell types crucial for USP26 action and to identify physiologically relevant substrates and genetic modifier(s) are needed. In conclusion, the present results clearly demonstrate that *Usp26* plays a critical role in spermatogenesis, and that the phenotypic outcomes associated with the *Usp26* mutation were dependent on genetic background.

## Methods

### Animals and ethics statement

B6D2F1 and ICR mice were purchased from Japan SLC (Shizuoka, Japan). B6D2F1 females were consecutively injected with pregnant mare serum gonadotropin (PMSG; ASKA Pharmaceutical, Tokyo, Japan) and human chorionic gonadotropin (hCG; ASKA Pharmaceutical) at a 46–48 h interval and then crossed with B6D2F1 males to collect fertilized eggs. Pseudopregnant mice were prepared by mating ICR females with vasectomized ICR males. All animal care and experiments were carried out in accordance with the Guidelines of Animal Experiments of Kitasato University and approved by the Institutional Animal Care and Use Committee of Kitasato University.

### Generation of Usp26 mutant mice

The guide RNA (gRNA) against the *Usp26* gene was designed using CRISPRdirect (https://crispr.dbcls.jp/)^43^. The sequence of the gRNA (Fig. 1B) was unique to the mouse genome and cloned into a pX330 plasmid (Addgene, Cambridge, MA, USA). The gRNA and *Cas9* mRNA were synthesized using the MEGAshortscript T7 *in vitro* transcription system (Thermo Fisher Scientific, Waltham, MA, USA) and dissolved in Opti-MEM (Thermo Fisher Scientific).

The gRNA and *Cas9* mRNA were introduced into fertilized eggs as previously described, with some modifications^44^. Briefly, fertilized eggs were isolated by removing cumulus cells via treatment with 30 µg/ml of hyaluronidase (Sigma–Aldrich, St Louis, MO, USA) in M2 medium, and then cultured in KSOM (ARK Resource, Kumamoto, Japan) at 37 °C and 5% CO_2_ until electroporation. The KSOM was replaced with Opti-MEM immediately prior to electroporation. Then, approximately 20 fertilized eggs were suspended in ice-cold Opti-MEM containing 200 ng/µl gRNA and 400 ng/µL *Cas9* mRNA and placed between two ice-cold platinum block electrodes connected to a CUY21Vivo-SQ pulse generator (BEX, Tokyo, Japan). Electroporation was carried out at room temperature at 30 V (3 ms ON + 97 ms OFF) seven times, and then the fertilized eggs were cultured in KSOM overnight at 37 °C and 5% CO_2_. The following day, 2-cell embryos were transferred into the fimbriae tubae of pseudopregnant mice, and pups (F0) were obtained via cesarean dissection.

### Staining of sperm and spermatids with peanut agglutinin

Sperm isolated from the cauda epididymis were fixed in 1% paraformaldehyde (PFA) in phosphate-buffered saline (PBS) for 15 min at 4 °C, washed once in PBS, and then stained with 4 µg/ml biotin-labeled PNA (J–Chemical; Tokyo, Japan) and 1 µg/ml fluorescein isothiocyanate (FITC)-labeled streptavidin (Biolegend, San Diego, CA, USA) in PBS. Subsequently, the nuclei were stained with 1 µg/ml 4′,6-diamidino-2-phenylindole (DAPI).

The testes were fixed in 4% PFA in PBS overnight at 4 °C, washed twice in PBS for 1 h at 4 °C, and then rotated in 10% and 20% sucrose solutions in PBS overnight at 4 °C. The dissected testes were embedded in OCT compound, frozen, and sectioned at 7 µm with a HYRAX C50 cryostat (Carl Zeiss, Jena, Germany). The sections were permeabilized with 0.1% Triton X-100 in PBS for 10 min, blocked for 1 h in 10% Blocking One reagent (Nacalai, Kyoto, Japan) in PBS at room temperature, and then incubated for 1 h at 4 °C with 4 µg/ml biotin-labeled PNA and 1 µg/ml FITC-labeled streptavidin in PBS. Nuclei were stained with 1 µg/ml DAPI. The stages of the seminiferous tubules were determined based on acrosomal and nuclear morphology as previously described^1^. Finally, the sperm and the testis sections were analyzed under a BX51 fluorescence microscope (Olympus, Tokyo, Japan) and photographs were taken using a DP73 digital CCD camera (Olympus).

### Histology and immunohistochemistry

The testes were fixed in Carnoy’s solution overnight at 4 °C, dehydrated, embedded in paraffin, and sectioned at 7 µm with a Micro HM325 microtome (Thermo Fisher Scientific). Next, the sections were stained with Mayer’s hematoxylin and eosin (HE; Fujifilm Wako Pure Chemical, Osaka, Japan) according to a standard protocol. The frozen sections of testes were prepared as described above, permeabilized with 0.1% Triton X-100 in PBS for 10 min, blocked for 1 h in 10% Blocking One reagent in PBS at room temperature, and then incubated overnight at 4 °C with primary antibodies. The following day, the sections were washed three times in PBS and incubated with secondary antibodies for 1 h at room temperature. A DAB staining kit (Nacalai) was used to visualize USP26 and the nuclei were stained with 1 µg/ml DAPI. Immunofluorescence was observed using a BX51 fluorescence microscope (Olympus) and photographs were taken using a DP73 digital CCD camera (Olympus).

### Chromosome spread

Chromosome spreads were prepared by a drying-down technique as described previously^45^. Immunolabeling was carried out as described above (*Histology and immunohistochemistry*).

### Western blot analysis

Testes lysates were prepared in a solution of 150 mM NaCl, 1% Nonidet P-40, 10 mM Tris–HCl (pH 8.0) containing EDTA–free protease inhibitor cocktail (Nacalai) and phenylmethylsulfonyl fluoride. The soluble materials were resolved on SDS–4–20% polyacrylamide gel (Bio-Rad Lab, Berkeley, CA, USA) and transferred onto a polyvinylidene difluoride membrane (Bio-Rad Lab). The filters were blocked with 5% skim milk in PBS containing 0.1% Tween20 and then sequentially incubated with primary and secondary antibodies. The blots were developed by Chemi–Lumi One Super (Nacalai) and signals were detected by LAS4000 (GE Healthcare, Chicago, IL, USA). For the detection of USP26, USP26 was immunoprecipitated from testes lysates with anti–USP26 antibody Ab2 (see *Antibodies*) and the precipitated proteins were subjected to Western blot analysis.

### Antibodies

The antibodies against mouse USP26 (Ab1 and Ab2) were generated using the procedure described below. First, a peptide corresponding to amino acids 95–114 (Ab1: CKRFLDKTSQGSIRPARSDER) or to amino acids 727–745 (Ab2: CSDDLDKKAKPTRKVDPTK) was conjugated to keyhole limpet hemocyanin and immunized to rabbit four times (Cosmo Bio, Tokyo, Japan). The anti–USP26 antibodies were purified on a peptide column.

The following antibodies were used for immunohistochemistry and immunocytochemistry: rabbit anti–mouse anti–USP26 Ab1 (7.1 µg/ml), mouse anti-Synaptonemal complex protein 3 (SYCP3; Cor 10G11/7 [ab97672], 1:500 dilution; Abcam, Cambridge, UK), rabbit anti–SYCP1 antibody (ab15090, 1:500 dilution, Abcam), rabbit anti–SYCP3 antibody (1:500 dilution, a gift from Dr. Shinichiro Chuma, Kyoto University)^46^, mouse anti–γH2AX antibody (3F2 [ab22551], 1:500 dilution, Abcam), mouse anti–MHL1 antibody (G168-15 [51-1327GR], 1:50 dilution, BD Bioscience, San Jose, CA, USA), CF568–conjugated goat anti–mouse IgG (H + L, 1:1000 dilution; Biotium, Fremont, CA, USA), and horse radish peroxidase (HRP)–conjugated donkey anti–rabbit IgG F(ab’)_2_ fragment (1:1,000 dilution; GE Healthcare).

The antibodies used for Western blot analysis were as follows: rabbit anti–mouse USP26 Ab2 (0.66 µg/ml), mouse anti–SYCP3 antibody (ab97672, 1:500 dilution, Abcam), rabbit anti–Equatorin antibody (EQ_70-83_, 1:4,000 dilution)^47^, mouse anti–β-actin antibody (AC-15 [A5441], 1:2,000 dilution, Sigma–Aldrich), HRP–conjugated donkey anti–rabbit IgG F(ab’)_2_ fragment (1:1,000 dilution; GE Healthcare) and HRP–conjugated sheep anti–mouse IgG F(ab’)_2_ fragment (1:1,000 dilution; GE Healthcare).

### Quantitative reverse-transcription polymerase chain reaction (qRT-PCR)

Total RNA was isolated using RNeasy Mini Kit (Qiagen, Valencia, CA, USA). Reverse transcription (RT) was performed using ReverTra Ace qPCR RT Kit (Toyobo, Osaka, Japan). Quantitative polymerase chain reaction (qPCR) was performed using FastStart Universal SYBR Green Master (ROX) (Roche, Rotkreuz, Switzerland) and analysed with CFX384 Real Time System (Bio-Rad Lab). The expression levels of each gene were normalized by expression of the housekeeping gene *Rplp0* (*Arbp*: *ribosomal protein large P0*). The primer sequences are listed in Table S1.

### TUNEL assay

The terminal deoxynucleotidyl transferase dUTP nick end labeling (TUNEL) assay was carried out using an *In situ* Apoptosis Detection Kit (Takara, Shiga, Japan) and the nuclei were counterstained with 1 µg/ml DAPI.

### Electron microscopic analysis

Adult testes and epididymides were fixed with 2.5% glutaraldehyde in 0.1 M phosphate buffer by perfusion through the left ventricle and immersed in the same fixative overnight. The tissues were cut into small pieces and fixed with 2% osmium tetroxide for 1 h. After dehydrated through a graded ethanol series, the tissues were embedded in Epon 812 (TAAB Laboratories Equipment, Berks, UK). One micrometer-thick sections were made for 1% toluidine blue (Wajdeck GmbH & Co, Munster, Germany) staining using an ultramicrotome (Ultracut E; Reichert-Jung, Vienna, Austria). Then, ultrathin sections were made and stained with uranyl acetate and lead citrate. The sections stained with toluidine blue were observed with an Olympus BX50 light microscope and analyzed using images captured by a RETIGA Exi FAST 1394 CCD (Charge-Coupled Device) camera (Qimaging, Surrey, BC, Canada). The ultrathin sections were analyzed using a JEOL JEM-1200 EX TEM (JEOL, Tokyo, Japan), as previously reported^48^.

## Supplementary information

Supplementary information file
