# Supplementary material for: Usp26 mutation in mice leads to defective spermatogenesis depending on genetic background
Source: Sci Rep. 2019 Sep 24;9:13757. doi: 10.1038/s41598-019-50318-6 (PMC6760205; doi:10.1038/s41598-019-50318-6)
Supplement: Supplementary file 1 — Supplementary information file [file 41598_2019_50318_MOESM1_ESM.pdf]

Usp26 mutation in mice leads to defective spermatogenesis depending on genetic background

Authors and affiliations:

Kohei Sakai[1], Chizuru Ito[2], Mizuki Wakabayashi[1], Satoko Kanzaki[1], Toshiaki Ito[1], Shuji Takada[3], Kiyotaka Toshimori[2,4], Yoichi Sekita[1] and Tohru Kimura[1,*]

[1] Laboratory of Stem Cell Biology, Department of Biosciences, Kitasato University School of Science, 1-15-1 Kitasato, Minami-ku, Sagamihara, Kanagawa 252-0373, Japan.

[2] Department of Functional Anatomy and Reproductive Biology and Medicine, Graduate School of Medicine, [4] Future Medicine Research Center, Chiba University Chiba University, 1-8-1, Inohana, Chuo-ku, Chiba, Chiba, 260-8670, Japan.

[3] Department of Systems BioMedicine, National Research Institute for Child Health and Development, 2-10-1, Okura, Setagaya-ku, Tokyo, 157-8535, Japan.

Correspondence:

[*] Tohru Kimura, PhD

Laboratory of Stem Cell Biology, Department of Biosciences, Kitasato University School of Science,

1-15-1 Kitasato, Minami-ku, Sagamihara, Kanagawa, 252-0373, Japan

Tel: +81-42-778-8864; Fax: +81-42-778-8864

E-mail: tkimura@ Kitasato-u.ac.jp

Figure S1

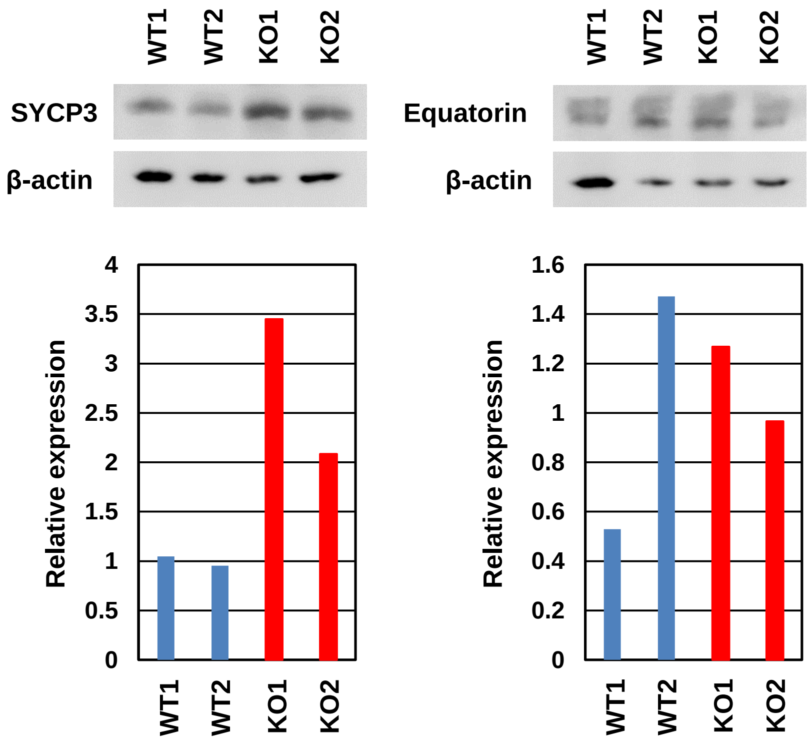
WT1
WT2
KO1
KO2
SYCP3
β-actin
Equatorin
β-actin
Relative expression
4
3.5
3
2.5
2
1.5
1
0.5
0
1.6
1.4
1.2
1
0.8
0.6
0.4
0.2
0

Figure S2

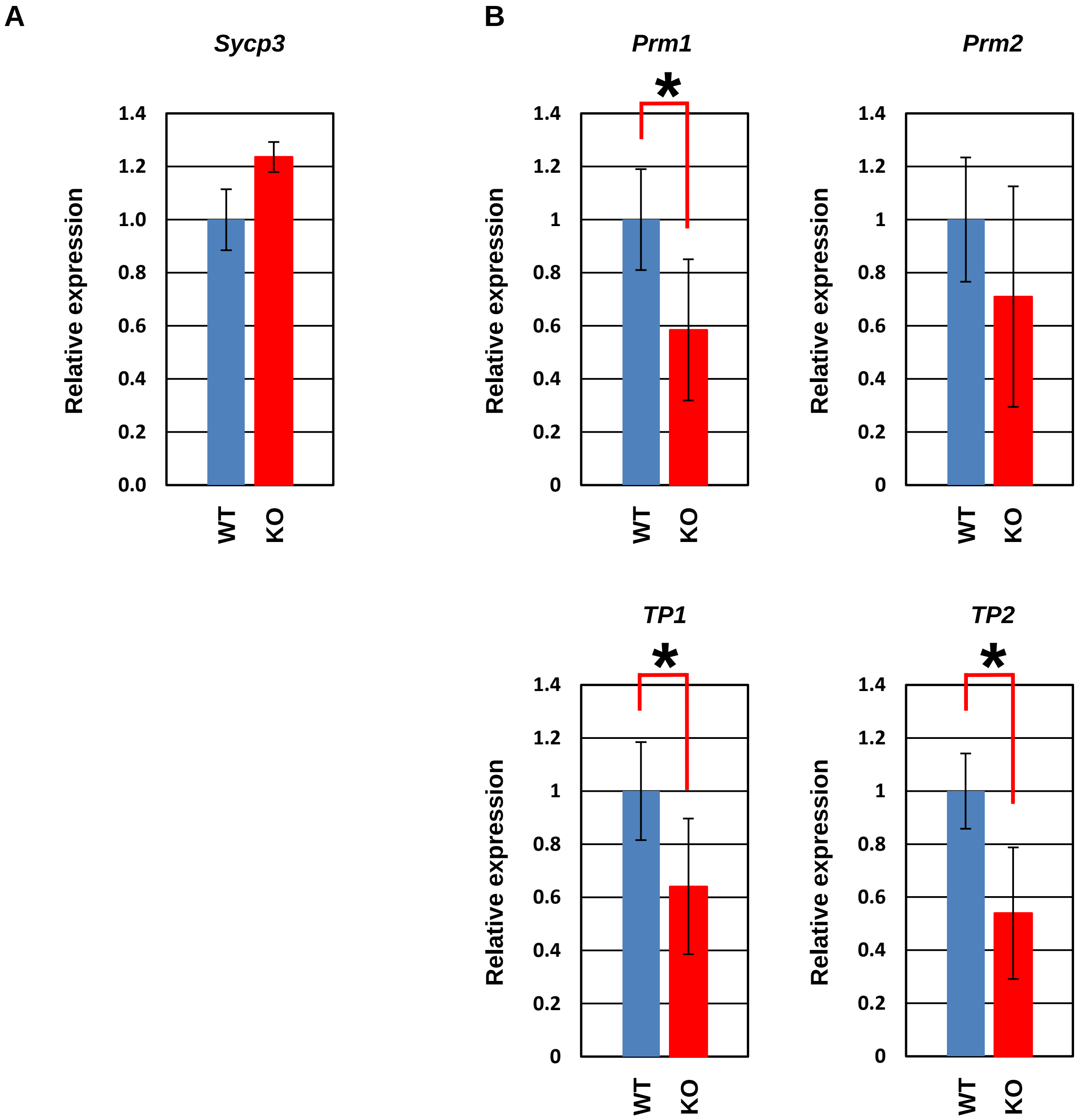
A
Sycp3
Relative expression
1.4
1.2
1.0
0.8
0.6
0.4
0.2
0.0
WT
KO
B
Prm1
*
Prm2
TP1
*
TP2
*
Relative expression
1.4
1.2
1
0.8
0.6
0.4
0.2
0
WT
KO

Figure S3

A

USP26

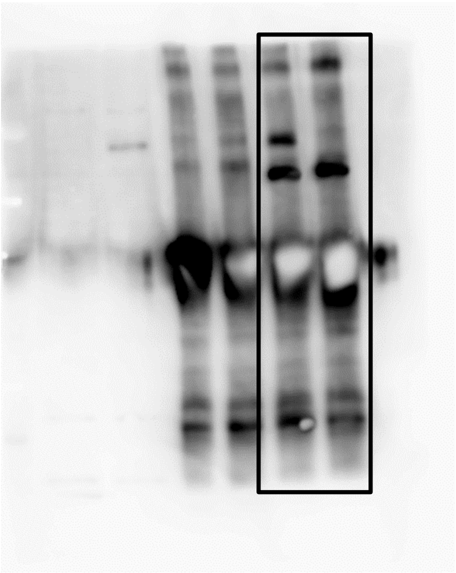

B

SYCP3 **Equatorin**

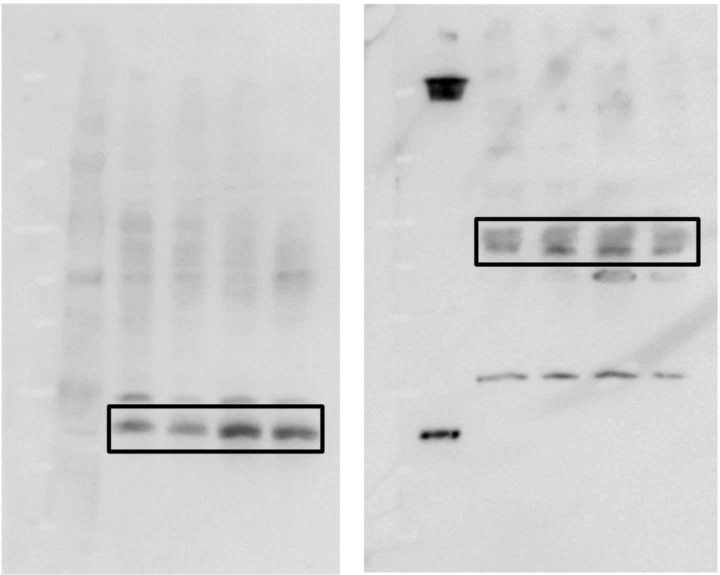

C

β-actin

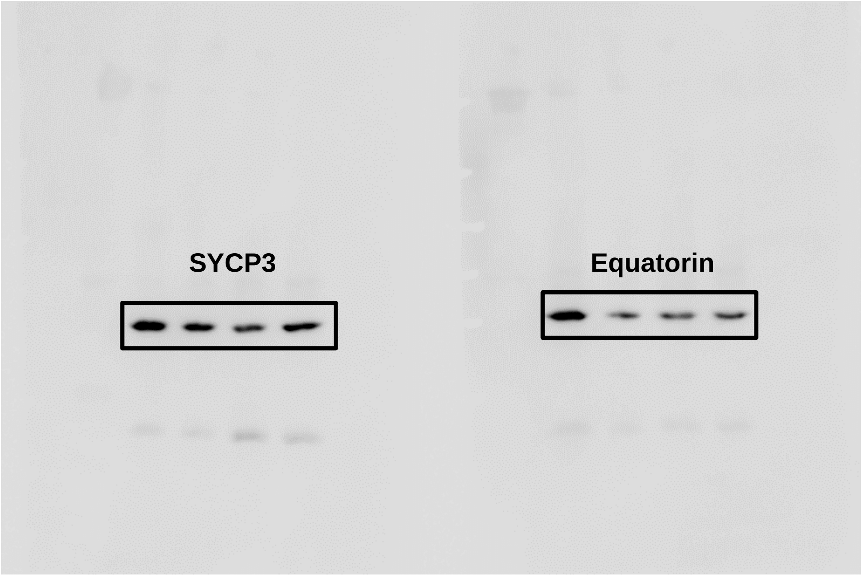

Supplementary Figure Legends

Figure S1. The protein levels of SYCP3 and Equatorin in *Usp26* mutant testis.

Testes lysates were subjected to Western blot analysis using anti–SYCP3 and anti–Equatorin antibodies. The band intensities were quantified using Image-J software and the expression levels were normalized by expression of β–actin.

Figure S2. Expression of spermatocyte and spermatid marker genes in *Usp26* mutant testis.

The mRNA levels of spermatocyte (A; *Sycp3*) and spermatid (B; *Prm1*, *Prm2*, *TP1*, *TP2*) marker genes in testes were quantified by RT-qPCR analysis. The expression levels were normalized by expression of the housekeeping gene *Rplp0* (*Arbp*: *ribosomal protein large P0*). Expression of *Prm1*, *TP1* and *TP2* was significantly decreased in mutant mice (Student's *t*-test; $^*P < 0.05$). The mean ± standard deviation is shown.

Figure S3. Uncropped blot images for Figure 1D (A) and Figure S2 (B, C).

Supplementary Tables

Table S1 Primer sequences for qRT-PCR

Gene	Sequence (5' to 3')	Sequence (5' to 3')
Sycp3	ATGGAAACTCAGCAGCAAGAG	TTTACTAAAGGCATGCCTCTTAGC
Prm1	TCAAAACTCCTGCGTGAGAA	GACAGGTGGCATTGTTCCTT
Prm2	CCTGTCCATTCTGCCTGGAG	ACTCAGATCTCGTGGCTCCT
TP1	GTCTTCAAACAACACGGGGC	CGAATTTCGTCACGACTGGC
TP2	TCCTGTGTCCATTTGATCCCA	AGTCTTCCAGGGTACAAGCT
Arbp	CAAAGCTGAAGCAAAGGAAGAG	ATTAAGCAGGCTGACTTGGTTG
